# Secondary damage in left-sided frontal white matter detected by diffusion tensor imaging is correlated with executive dysfunction in patients with acute infarction at the ipsilateral posterior corona radiata

**DOI:** 10.1186/s40001-014-0044-x

**Published:** 2014-08-20

**Authors:** Chuo Li, Chao Dang, Gang Liu, Li Chen, Jian Zhang, Jingjing Li, Zilin Ou, Yusheng Zhang, Anding Xu

**Affiliations:** 1 Department of Neurology, Guangzhou Number 8 People’s Hospital, Guangzhou Medical University, 8, Huaying Road, Guangzhou 510440, China; 2 Department of Neurology and Stroke Center, the First Affiliated Hospital, Sun Yat-Sen University, 58, Zhongshan Road 2, Guangzhou 510080, China; 3 Department of Neurology, the First Affiliated Hospital, Jinan University, West 613, Huangpu Road, Guangzhou 510630, China

**Keywords:** Cerebral infarction, Executive dysfunction, Diffusion tensor imaging

## Abstract

**Background:**

Executive dysfunction has been observed in patients with left-sided anterior corona radiata infarction. However, whether left-sided posterior corona radiata infarction could cause executive dysfunction is unclear. Also, whether secondary damage in the left frontal white matter following ipsilateral posterior corona radiata infarct is causal or not and contributes to the occurrence and development of executive dysfunction, is still uncertain.

**Methods:**

Twelve patients with posterior corona radiata infarction underwent diffusion tensor imaging (DTI) and an executive functional assessment at week 1 (W1), week 4 (W4), and week 12 (W12) after onset. Color duplex sonography and Transcranial Duplex Scanning (TCD) were performed at W1 and W12. Twelve healthy volunteers of similar ages and educational histories were examined as controls and assessed once.

**Results:**

In the patients, we observed an increased mean diffusivity (MD) and a decreased fractional anisotropy (FA) in the left frontal white matter from W1 to W12. There were no significant changes in cerebral blood flow in patients between W1 and W12 according to the result of Color duplex sonography and TCD. Patients showed progressively impaired executive function during 12 weeks. Significant correlations were found between increased MD and decreased FA in the left frontal white matter with impaired degree of executive function.

**Conclusions:**

This study demonstrates that DTI detected secondary damage in left-sided frontal white matter in patients with acute infarction at the ipsilateral posterior corona radiata. This change may be correlated with executive functional changes in these patients.

## Background

The term ‘executive functions’ is used to describe high-order cognitive processes which include control, integration, organization and maintenance of other cognitive abilities [1]. Executive dysfunction might predict poor functional outcome of patients with stroke [2]. The frontal lobes have extensive connections with portions of the limbic system, the basal ganglia, and the thalamus. The frontal-subcortical white matter connections as well as the frontal lobe are well known to be functionally critical in the control of executive function [3]. Lesions of not only the prefrontal cortex but subcortical areas result in executive dysfunction [4],[5]. In particular, damage of frontal white matter, which is an important region in frontal-subcortical circuitry, results in this executive dysfunction [6].

Recently, accumulating studies have shown histopathological and radiological changes occurring in nonischemic remote brain regions that have synaptic connections with the primary lesion site. For example, after cerebral infarction in the middle cerebral artery (MCA) territory, neuronal and/or axonal degeneration, loss, dysfunction and gliosis have been found in the ipsilateral thalamus, substantia nigra, and distal pyramidal tract, all of which were remote from the MCA territory [7]–[12]. The corona radiata is the core of the hemispheric white matter, and its infarction may compromise the integrity of the frontal-subcortical white matter connections. A study of patients with stroke scanned by traditional magnetic resonance imaging (MRI) showed that ischemic infarctive lesions affecting the left-sided anterior corona radiata caused executive dysfunction [4]. The disruption of frontal-subcortical circuitry was considered to be responsible for this executive dysfunction. However, whether the left-sided posterior corona radiata infarction could cause executive dysfunction is unclear. Moreover, the underlying pathogenesis, especially whether secondary damage in other areas, such as frontal white matter in frontal-subcortical circuitry, due to anterograde and/or retrograde degeneration following focal corona radiata infarction, is a causal phenomenon and is involved in the dysfunction, is still uncertain.

The recent development of diffusion tensor imaging (DTI), an MRI technique, has shown promise for detecting microstructural changes which are not identified by traditional MRI. For example, progressive decreased fractional anisotropy (FA) values in the pyramidal tract, proximal and distal to a subcortical cerebral infarct or pontine infarct over 1 to 12 weeks, were found; this means important loss of structural components in the pyramidal tract above and below the primary infarcts [11],[12]. In patients with ischemic leukoaraiosis (ILA), increased mean diffusivity (MD) and decreased FA were observed in the frontal and occipital white matter which illustrated axonal loss in the regions [6]. These studies suggest potential secondary damage in white matter might be observed by DTI after corona radiata infarction.

In this study we tested the hypothesis that there is axonal loss in the left-sided frontal white matter in patients following acute ipsilateral posterior corona radiata infarction as revealed by DTI. We also examined correlations of the DTI parameters with executive function in these patients.

## Methods

### Participants

The research protocol was approved by the Medical Ethical Committee of the First Affiliated Hospital, Sun Yat-Sen University, and informed consent was obtained from all participants. We selected 12 consecutive patients (7 male, 5 female) between October 2008 and May 2013 in the Department of Neurology and Stroke Center, the First Affiliated Hospital, Sun Yat-Sen University, within 7 days of a focal infarct in the left posterior corona radiata, without any other signal abnormalities on T1-weighted fluid attenuated inversion recovery (T1-FLAIR) and T2-weighted images. Patients with ILA, significant cerebral atrophy, unstable vital signs or a history of central nervous system disorders were excluded. Demographic characteristics and vascular risk factors were recorded in each patient. All patients were investigated with conventional MRI, DTI and executive functional assessments at 1 week (6.3 ± 1.1 days, W1), 4 weeks (30.1 ± 2.8 days, W4), and 12 weeks (92.8 ± 5.8 days, W12) following a pre-defined protocol. Infarct volume was estimated from the T2-weighted FLAIR images at W12. For every patient, we looked for a control of the similar age (±3 years) and educational history (±2 years) with no current or previous neurological or psychiatric disease. Twelve controls were recruited over the same period and examined once by DTI and an executive functional assessment.

### MRI protocol

MRIs were performed using a 1.5-tesla MRI system (Signa General Electric Medical Systems, Milwaukee, WI, USA) equipped with gradient hardware allowing up to 23 mT/m. T1-FLAIR, fast spin echo T2-weighted imaging, traditional FLAIR and DTI were performed. Typical acquisition parameters of conventional MRI were: T1-FLAIR (6 mm thick, 2 mm gap, TR 1,475 ms/TE 21.3 ms/TI 750.0 ms), T2-FLAIR (3 mm thick, 0 mm gap, TR 8,000 ms/TE 120.0 ms/TI 2,200 ms). DTI was obtained with an echo planar imaging (EPI) sequence (3 mm thick, 0 mm gap, TR 10,000/TE 100 ms, NEX = 1, matrix 128 × 128, field of view 24 × 24 cm). DTI was acquired with a b factor of 1,000 s/mm^2^ and diffusion-sensitive gradients were applied along 15 gradient directions. In addition, a reference image without diffusion weighting (b = 0 s/mm^2^) was acquired. Fifteen repeats were acquired and averaged to improve signal to noise ratio. The scanning baseline was parallel to the antero-posterior commissural (AC-PC) line. Axial slices were obtained using a fast spin echo T2 (3 mm thick, 0 mm gap, TR 5,660 ms/TE 107.7 ms).

### Image post-processing

**Figure 1:**
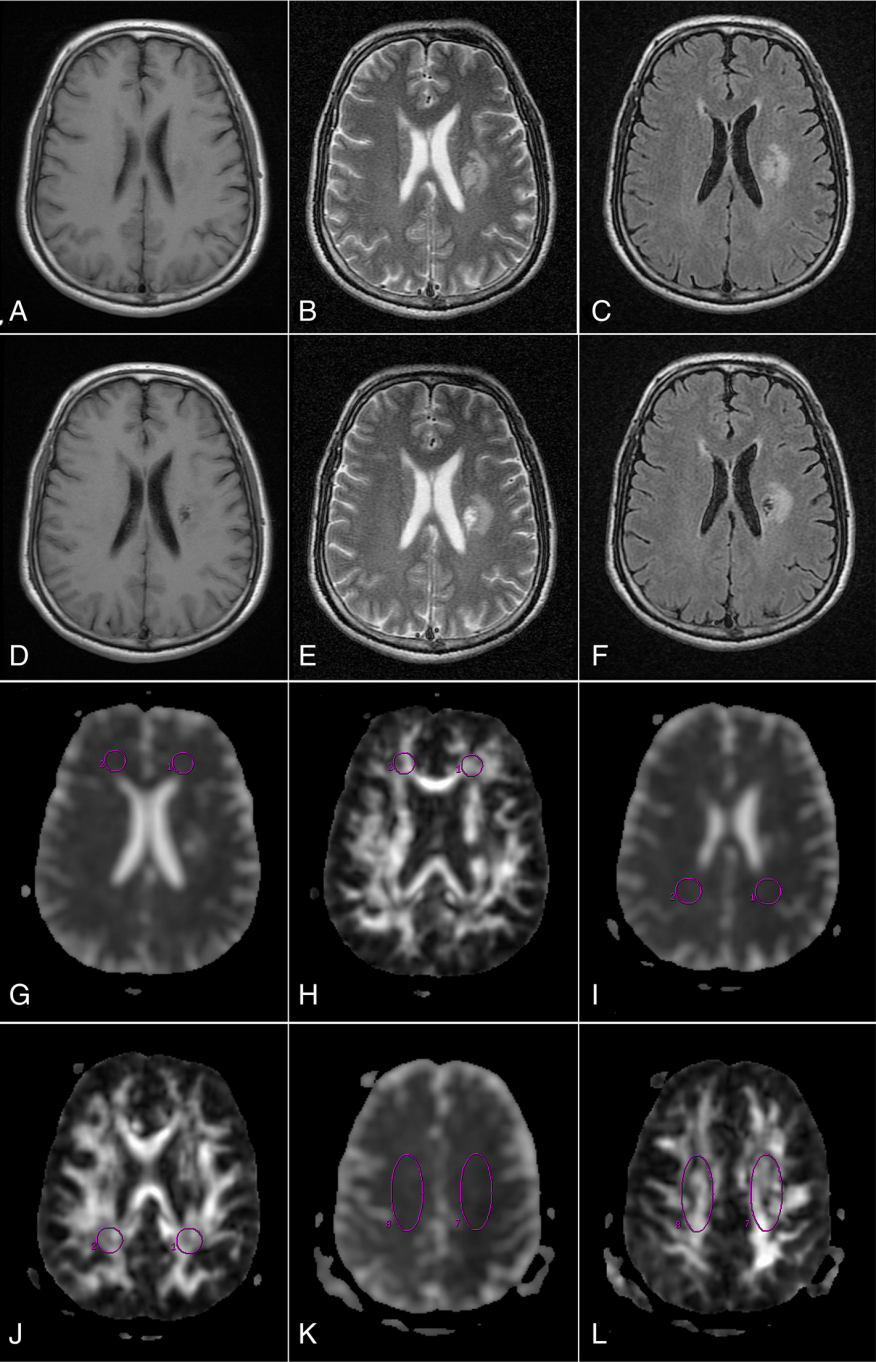
**Ischemic lesion and regions of interest (ROIs) are shown in the traditional magnetic resonance and diffusion tensor magnetic resonance images from a patient with acute infarction at the left posterior corona radiata. (A)** T1-FLAIR obtained at week 1 (W1) after stroke show a hypointense area at the left posterior corona radiata. **(B)** T2-weighted image and **(C)** T2-FLAIR images obtained at W1 show a hyperintense area at the left posterior corona radiata. **(D)**, **(E)** and **(F)** show the lesion at W12. **(G)**, **(I)** and **(K)** mean diffusivity images and **(H)**, **(J)** and **(L)** fractional anisotropy images show ROIs in the left and right frontal white matter, posterior periventricular white matter and centrum semiovale.

An experienced radiographer carried out image post-processing without knowing the purpose of the study. The DTI data were transferred to a workstation and spatially filtered using a median 3 × 3 filter. Acquiring the DTI data with the dual-echo EPI sequence and ramp sampling considerably reduced, but did not eliminate, the geometric distortions. We corrected the residual distortions by registering the diffusion-weighted imaging with the reference image using the two-dimensional module of the automated image registration package (eight-parameter with perspective) [13]. With this correction, the DTI parameters were calculated on a pixel-by-pixel basis. Using ADW4.2 Software (Signa General Electric Medical Systems, Milwaukee, WI, USA), regions of interest (ROIs) were manually delineated in the left and right frontal white matter (an area of 150 mm^2^) defined as quasi-circular, the white matter around the posterior horn (an area of 224 mm^2^) defined as quasi-circular, the centrum semiovale defined as ellipse (an area of 780 mm^2^) on a slice from the FLAIR images and then transferred to the DTI image on the same slice (Figure 1). The thalami were also observed at the same times. According to the methods used by Hervé *et al*., the medial boundaries of the thalami were verified by the limits of cerebrospinal fluid-containing ventricle on T2-FLAIR, and the lateral limits were defined by the internal capsule on FA maps [14]. DTI maps were calculated at W1 and on follow-up scans. The quantitative MD (derived from the trace of the diffusion tensor, MD = trace (D)/3) and FA data in frontal white matter obtained at different time points were analyzed.

### Color duplex sonography and transcranial duplex scanning (TCD)

The examinations with Color duplex sonography of the extracranial carotid arteries were performed with the patients at W1 and W12 and controls at recruitment. All measurements were performed by the angiologist who was blinded for the clinical status of the study patients and controls. The internal carotid artery (ICA) was explored using a 10-MHz linear-array transducer (GE LOGIQ5 ultrasound system, Milwaukee, WI, USA). The subjects were in the supine position with their heads slightly elevated and turned 25° to 40° to the contralateral side for the ICA measurements. Peak systolic velocity (PSV) and end diastolic velocity (EDV) were measured 1 to 2 cm above the carotid bulb in the ICAs. Intima-media thickness (IMT) and luminal diameter (DIA) of the ICAs were measured on magnified B-mode. The distance between the leading edge of the luminal echo and the leading edge of the media/adventitia echo was measured as the IMT.

The patients underwent TCD scanning at W1 and W12 and controls were scanned at recruitment. Bilateral mean flow velocity was continuously and simultaneously monitored by a 2-MHz phased array transducer (Philips SONOS 5500, Eindhoven, the Netherlands) by the angiologist who was not familiar with the clinical status of the study patients and controls. The highest signal was sought at a depth ranging (anterior cerebral artery: 61 to 75 mm; middle cerebral artery: 46 to 55 mm, posterior cerebral artery: 56 to 70 mm) through the temporal bone window. Mean flow velocity was calculated and recorded automatically when the ‘record’ function of the Doppler instrument was activated.

### Assessment of executive dysfunction

A ‘brief executive assessment’ was also conducted which include four tests often used clinically: the Trail making B-A test, the Verbal fluency assessment, the Digit span backwards test and the Digit symbol test. This has been considered to be a sensitive tool for detecting deficits in patients and distinguishing these deficits from the cognitive effects of healthy aging [6],[15]. Because elderly Chinese patients are unfamiliar with the English language, the Trail making B-A test (Chinese version) and Verbal fluency test (Chinese version) were applied [6],[16],[17]. The Mini-Mental State Examination (MMSE) was also applied because it is often used clinically as a standard measure of dementia severity.

### Statistical analysis

MD and FA values obtained at W1, W4, and W12 in patients were first compared with those of controls with a two-tailed Student’s *t*-test. Executive assessments were compared between the patient and the control groups using a Student’s *t*-test. These parameters and clinical scores then were analyzed using Fisher’s Least Significant Difference (LSD) test for multiple testing. The Color duplex sonography and TCD parameters obtained at W1 and W12 were compared with a two-tailed Student’s *t*-test. Spearman rank correlation analysis was used to determine associations between changes in brief executive assessment scores and the DTI parameter changes in left frontal white matter and thalamus. The changes in executive function scores over time and the DTI parameter changes were defined as follows:

ΔExecutive scores or DTI Parameter values = Value measured at W12 − value measured at W1

## Results

### Participants

Twelve patients (seven male and five female) and twelve controls (seven male and five female) were recruited in the study. All patients and controls were right-handed. Each patient had one or more vascular risk factor (Table 1).

**Table 1:** Patient demographics and clinical data

**Number**	**Age (years)**	**Gender**	**Educational history (years)**	**Vascular factors**	**Volume of lesion (mm**^**3**^**)**
1	59	M	8	Hypertension	11,160
2	67	F	6	Hypertension, hypercholesterolemia, diabetes mellitus	14,000
3	66	M	18	Hypertension, tobacco use, overweight	13,300
4	57	F	7	Hypertension, diabetes mellitus	9,690
5	66	M	8	Hypertension, diabetes mellitus	6,790
6	65	M	6	Tobacco use	8,450
7	46	F	11	Hypercholesterolemia	7,370
8	69	F	6	Hypertension	10,810
9	63	M	8	Hypertension, tobacco use	16,200
10	48	F	8	Hypertension	9,660
11	65	M	12	Hypertension, diabetes mellitus, tobacco use, overweight	12,150
12	50	M	5	Hypertension, diabetes mellitus	8,810

F: female; M: male; FLAIR: fluid attenuated inversion recovery. The volume of the infarcted lesion was calculated on T2-FLAIR images at week 12 (W12). Controls were matched for age and educational history.

### MRI data

Participants completed all traditional MRI and DTI examinations. Within the first week from stroke onset, T2-weighted and T2-FLAIR images showed focal hyperintensity confined to the left-sided posterior corona radiata in each patient (Figure 1B and C), and there was no abnormal signal in other regions. At W12, no signal abnormality was detected on T1, T2-weighted, and T2-FLAIR images outside the territory of the left posterior corona radiata (Figure 1D, E and F). The infarct volume varied from 6,790 to 16,200 mm^3^ (10,699 ± 2,818 mm^3^) on T2 images at W12. No definite abnormal signals were observed in controls.

In controls, in the absence of any significant difference between the left and right regions for MD (mean values: frontal white matter 0.804 ± 0.109 × 10^−3^ mm^2^/s and 0.809 ± 0.108 × 10^−3^ mm^2^/s; posterior periventricular white matter 0.824 ± 0.099 × 10^−3^ mm^2^/s and 0.816 ± 0.088 × 10^−3^ mm^2^/s; centrum semiovale 0.764 ± 0.093 × 10^−3^ mm^2^/s and 0.771 ± 0.095 × 10^−3^ mm^2^/s; thalamus 0.836 ± 0.069 × 10^−3^ mm^2^/s and 0.840 ± 0.075 × 10^−3^ mm^2^/s, respectively, all *P* > 0.05) and FA (mean values: frontal white matter 0.421 ± 0.057 and 0.433 ± 0.102; posterior periventricular white matter 0.451 ± 0.057 and 0.449 ± 0.056; centrum semiovale 0.392 ± 0.063 and 0.397 ± 0.065; thalamus 0.345 ± 0.051 and 0.343 ± 0.049, respectively, all *P* > 0.05), the data obtained in the left regions in controls were selected to compare those values in patients.

MD and FA values at W1 in the left and right regions (frontal white matter, posterior periventricular white matter, centrum semiovale and thalamus) were not significantly different compared with those of controls (*P* > 0.05). There was a significant increase of MD and decrease of FA in the left frontal white matter from W1 to W12 (*P* < 0.05). No obvious MD or FA value changes were found in the right frontal white matter (*P* > 0.05). There were no significant differences of MD and FA in the left and right posterior periventricular white matter and centrum semiovale from W1 to W12 (*P* > 0.05). In the thalamus, a significant increase of MD in the left thalamus was found between W1 and W12 (*P* < 0.05), and no obvious MD value changes were found in right thalamus (*P* > 0.05). There was no significant difference of the FA values in left and right thalami between W1 and W12. No significant differences of MD and FA in the posterior periventricular white matter or centrum semiovale were detected from W1 to W12 (*P* > 0.05) (Table 2).

**Table 2:** Magnetic resonance imaging (MRI) parameter values in frontal white matter, posterior periventricular white matter, centrum semiovale and thalamus in patients and controls (mean ± SD)

**Region**	**MD (×10**^**−3**^ **mm**^**2**^**/s)**			**FA**		
	**W1**	**W4**	**W12**	**W1**	**W4**	**W12**
Patients						
LFWM	0.824 ± 0.095^a^	0.815 ± 0.080	0.904 ± 0.107^bc^	0.388 ± 0.056^a^	0.374 ± 0.062	0.313 ± 0.054^bc^
RFWM	0.825 ± 0.089	0.821 ± 0.088	0.831 ± 0.084	0.387 ± 0.062	0.385 ± 0.063	0.395 ± 0.063
LPPWM	0.837 ± 0.102	0.836 ± 0.096	0.839 ± 0.087	0.452 ± 0.062	0.447 ± 0.061	0.431 ± 0.063
RPPWM	0.853 ± 0.114	0.835 ± 0.094	0.822 ± 0.085	0.444 ± 0.053	0.451 ± 0.059	0.447 ± 0.053
LCS	0.766 ± 0.112	0.754 ± 0.087	0.729 ± 0.104	0.387 ± 0.058	0.381 ± 0.060	0.362 ± 0.055
RCS	0.772 ± 0.110	0.765 ± 0.098	0.783 ± 0.105	0.383 ± 0.059	0.391 ± 0.062	0.393 ± 0.064
L thalamus	0.846 ± 0.080^a^	0.830 ± 0.097	0.949 ± 0.065^bc^	0.338 ± 0.038	0.334 ± 0.037	0.323 ± 0.032
R thalamus	0.839 ± 0.788	0.840 ± 0.081	0.841 ± 0.083	0.344 ± 0.042	0.344 ± 0.043	0.342 ± 0.040
Controls						
LFWM	0.804 ± 0.109			0.421 ± 0.057		
LPPWM	0.824 ± 0.099			0.451 ± 0.057		
LCS	0.764 ± 0.093			0.392 ± 0.063		
L thalamus	0.836 ± 0.069			0.345 ± 0.051		

*Abbreviations*: FA, fractional anisotropy; LCS, left centrum semiovale; LFWM, left frontal white matter; LPPWM, left posterior periventricular white matter; LCS, left centrum semiovale; L thalamus, left thalamus; MD, mean diffusivity; R thalamus, right thalamus; RCS, right centrum semiovale; RFWM, right frontal white matter; RPPWM, right posterior periventricular white matter; W1, the first week; W4, the fourth week; W12, the 12th week.

^a^*P* < 0.05, LSD test, comparison between W1 and W12; ^b^*P* < 0.05, Student’s *t*-test, comparison between the control and patient groups; ^c^*P* < 0.05, Student’s *t*-test, comparison in the left and right region of patients at W12.

The controls were examined only once when they were recruited.

### Color duplex sonography and TCD data

The Color duplex examination of luminal diameter and IMT measurements were performed in all the ICAs of the subjects. The mean values of all data were given in Table 3. We did not find any differences of IMT, DIA, PSV and EDV detected at W1 and W12 in patients (all *P* > 0.05).

**Table 3:** Ultrasound parameters of the left and right internal carotid artery (mean ± SD)

**Group**	**Left IMT (mm)**		**Right IMT (mm)**		**Left DIA (mm)**		**Right DIA (mm)**		**Left SPV (cm/s)**		**Right SPV (cm/s)**		**Left EDV (cm/s)**		**Right EDV (cm/s)**	
	**W1**	**W12**	**W1**	**W12**	**W1**	**W12**	**W1**	**W12**	**W1**	**W12**	**W1**	**W12**	**W1**	**W12**	**W1**	**W12**
Patients	1.21 ± 0.13	1.22 ± 0.14	1.18 ± 0.09	1.18 ± 0.10	4.86 ± 0.63	4.84 ± 0.61	4.97 ± 0.55	5.02 ± 0.58	61 ± 9.8	65 ± 11.1	58 ± 8.7	60 ± 9.4	24.3 ± 3.8	24.5 ± 3.7	23.9 ± 3.7	24.1 ± 3.8
Controls	1.02 ± 0.19		1.04 ± 0.22		5.20 ± 0.68		5.37 ± 0.73		52 ± 8.5		53 ± 8.3		23.5 ± 5.6		24.0 ± 6.1	

*Abbreviations*: DIA, luminal diameter; EDV, end diastolic velocity; IMT, intima-media thickness; PSV, peak systolic velocity; W1, the first week, W12, the 12th week.

The controls were examined only once when they were recruited.

All subjects had accessible cranial window for TCD and underwent 0.5 to 1 hour of TCD monitoring. Mean flow velocity values (MFV) were shown in Table 4. There were no statistically significant differences of MFV detected in anterior cerebral artery (ACA), MCA and posterior cerebral artery (PCA) at W1 and W12 in patients (all *P* > 0.05).

**Table 4:** Values of the mean flow velocity (cm/s) in the left and right anterior cerebral artery (ACA), middle cerebral artery (MCA) and posterior cerebral artery (PCA) (mean ± SD)

**Group**	**Left ACA**		**Right ACA**		**Left MCA**		**Right MCA**		**Left PCA**		**Right PCA**	
	**W1**	**W12**	**W1**	**W12**	**W1**	**W12**	**W1**	**W12**	**W1**	**W12**	**W1**	**W12**
Patients	55.17 ± 9.53	53.85 ± 9.94	49.74 ± 9.13	50.65 ± 8.06	58.94 ± 15.42	60.07 ± 11.26	63.12 ± 13.63	63.26 ± 12.65	38.8 ± 10.76	37.21 ± 8.10	38.17 ± 12.40	38.99 ± 13.02
Controls	46.62 ± 7.05		47.81 ± 8.89		64.95 ± 9.35		62.88 ± 5.40		37.20 ± 5.65		36.97 ± 6.13	

*Abbreviations*: W1, the first week; W12, the 12th week.

The controls were examined only once when they were recruited.

### Cognitive deficit

There were no significant differences in MMSE, Verbal fluency assessment and Digit span backwards test between patients and controls at W1. The significant difference of Trail making B-A and Digit symbol tests were found between patients and controls at W1. Mean scores on the Trail making B-A test, Verbal fluency assessment, Digit span backwards and Digit symbol tests for patients were significantly changed between W1 and W12 (*P* < 0.05). There was statistical difference in the presence of Trail making B-A, Verbal fluency assessment and Digit symbol tests between patients and controls at W4 and W12, and Digit span backwards test at W12 (Table 5).

**Table 5:** Cognitive functional scores of patients and controls (mean ± SD)

	**Patients**			**Controls**
	**W1**	**W4**	**W12**	
MMSE	26.8 ± 1.6	25.4 ± 2.3	25.0 ± 1.7	29.3 ± 1.5
Trail making B-A test	160.3 ± 54.7^ab^	181.8 ± 52.1^b^	224.1 ± 55.1^b^	77.2 ± 33.3
Verbal fluency assessment	40.8 ± 10.3^a^	33.9 ± 11.0^b^	26.9 ± 11.0^b^	42.4 ± 8.1
Digit span backwards test	7.3 ± 1.5^a^	6.3 ± 1.4	5.7 ± 1.3^b^	7.0 ± 1.7
Digit symbol test	32.6 ± 6.5^ab^	26.6 ± 6.5^b^	22.3 ± 4.9^b^	45.6 ± 9.2

*Abbreviations*: MMSE, Mini-Mental State Examination; W1, within the first week; W4, the fourth week; W12, the 12th week.

^a^*P* < 0.05, LSD test, comparison between W1 and W12 in patients; ^b^*P* < 0.05, Student’s *t*-test, comparison between the control and patient groups.

### Correlations between DTI and executive function in patients

**Figure 2:**
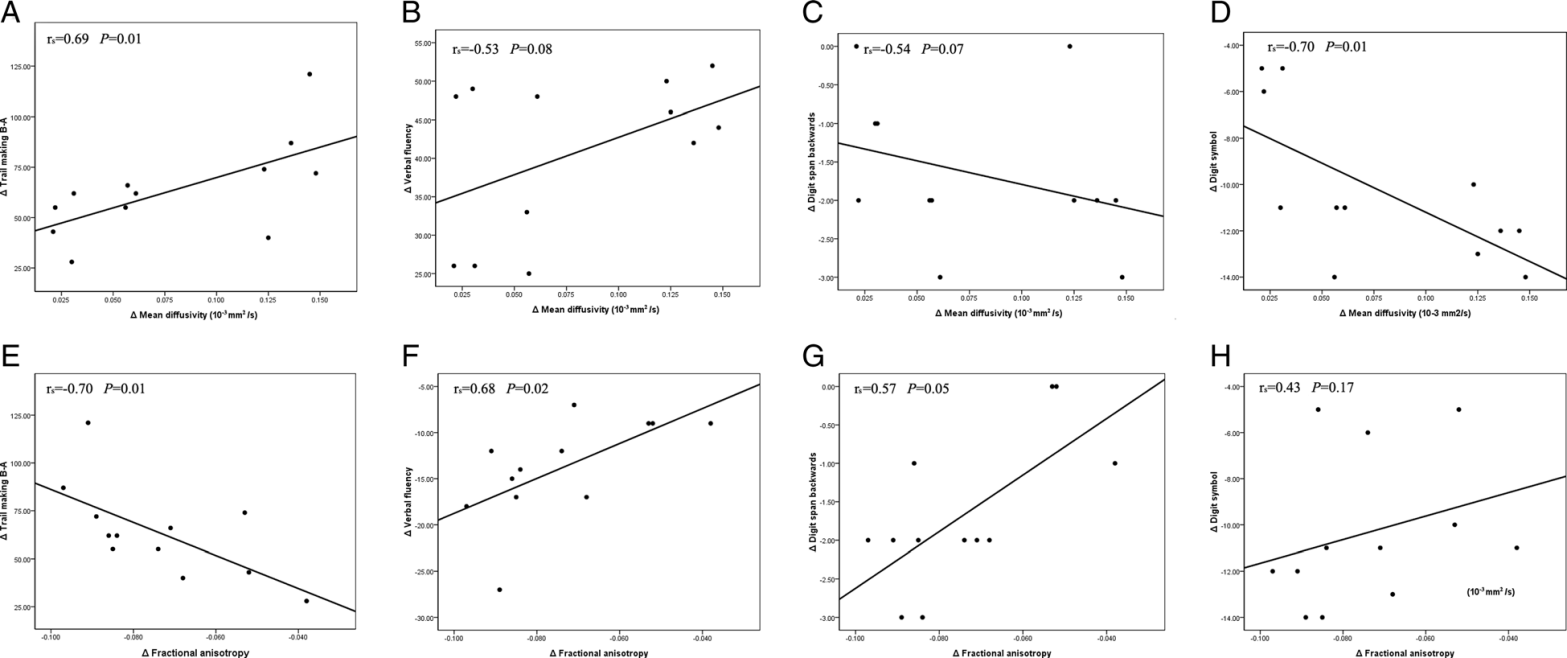
**Scatter plot with lines of best fit showing the relationship between the change values of diffusivity (mean diffusivity and fractional anisotropy) in the left frontal white matter and the change values of executive function (trail making B-A, verbal fluency assessment, digit span backwards and digit symbol tests) in patients from W1 to W12.** A-H show the relationship between Δmean diffusivity and Δtrail making B-A **(A)**, Δverbal fluency **(B)**, Δdigit span backwards **(C)**, Δdigit symbol **(D)**, and between Δfractional anisotropy and Δtrail making B-A **(E)**, Δverbal fluency **(F)**, Δdigit span backwards **(G)**, Δdigit symbol **(H)**.

As illustrated in Figure 2, the Spearman rank correlational analyses showed that the MD and FA changes in the left frontal white matter correlated with the executive function changes in patients. There were only weak correlations between the MD changes in the left thalamus and Trail making B-A test (Spearman coefficient of rank correlation r_s_ = 0.488, *P* = 0.098) and Digit symbol test (Spearman coefficient of rank correlation r_s_ = −0.552, *P* = 0.063).

## Discussion

To the best of our knowledge, this is the first prospective study using DTI to observe secondary changes in the left frontal white matter remote from the ipsilateral posterior corona radiata.

Previous studies have shown that focal cerebral infarcts can lead to remote tissue alterations within connected regions related to Wallerian degeneration [8],[10],[18]–[21]. Because computed tomography (CT) and traditional MRI are insufficiently sensitive and difficult to quantify the microstructural changes, DTI was used in this study. DTI is able to characterize brain tissue structure by measurement of water diffusion, expressed as two indices, MD and FA. MD is a parameter of magnitude of average molecular motion considered in all directions. FA is a quantitative measure of directional bias in the diffusion profile. A significant increase of MD and decrease of FA in the left frontal white matter was found between W1 and W12, but there were no significant changes between W1 and W4, and between W4 and W12. The abnormal diffusion observed suggest that the secondary damage is cumulative from W1 to W12. Such changes in MD and FA in the frontal white matter have also been described in patients with ILA, which is suggestive of axonal structural loss in the regions [6]. Also, the increased MD and decrease of FA may be correlated with executive functional changes.

Frontal-subcortical circuit was defined as including the frontal cortex, caudate, pallidum, thalamus, genu of internal capsule, anterior internal capsule, anterior corona radiata, and anterior centrum semiovale [3]. In this study, executive dysfunction was found in the patients with left posterior corona radiata infarction. A possible explanation for this is that the region itself is a structure in the executive functional circuit. Another more possible mechanism of the executive changes in the patients is secondary degeneration due to the disruption of axons in the frontal white matter that links the frontal cortex to other structures in the executive functional circuit. The correlation of MD and FA alteration in the left posterior white matter with executive performance supports this hypothesis.

Our previous study has shown that gray matter volumes decreased significantly in the ipsilateral supplementary motor area and contralateral insula from W1 to W12 in patients with undergoing acute subcortical infarction. The changes of gray matter volumes in the ipsilesional supplementary motor area correlated with the changes of motor function and activities of daily living [22]. In the present study, a significant increase of MD in the left thalamus was found over time, which is suggestive of an important loss of thalamic structural components. Within the thalamus, secondary damage has been also reported by Hervé *et al*. and our team in patients after an acute isolated MCA territory infarction [8],[14]. A weak correlation between the MD changes in the left thalamus and executive function was found in this study, which means secondary damage of the left thalamus is possibly involved in inhibiting executive function. The lack of FA changes may be related to the absence of isolated bundles of parallel fibers within the thalamus [14]. We failed to find significant diffusion changes in the posterior periventricular white matter or centrum semiovale. It is possible that only the most significant diffusion changes were detected by our method and more widespread secondary damages occur in other areas. Longer follow-up and more sophisticated image analysis techniques may be required for determining secondary damage in other regions remote to an infarction.

In our patients, there was no abnormal signal in the frontal area at W1 and W12, which means no new ischemic lesions exist in the frontal area between W1 and W12. There was also no significant change of blood flow in the frontal area between W1 and W12 in patients as measured by the evaluation of carotid artery stenosis and hemodynamics of ACA, MCA and PCA performed with Color duplex sonography and TCD, suggesting that frontal dysfunction would not be attributed to potential hemodynamic changes.

Vataja *et al*. reported significant executive dysfunction in patients with anterior corona radiata infarction, but failed to find executive change in patients with posterior corona radiata infarction [4]. In this study, however, we observed a poor executive performance in patients with posterior corona radiata infarction. The difference between our study and that of Vataja *et al*. was that our study included patients without white matter lesions (WMLs) and cerebral atrophy. WMLs were described radiologically as LKA in periventricular white matter, and subcortical, deep, watershed areas. Executive dysfunction has been found to associate with the degree of WMLs assessed by MRI, DTI or MR spectroscopy [6],[23]–[26]. Cerebral atrophy was also related to executive dysfunction [26],[27]. In this study, executive change might be observed more easily owing to posterior corona radiata while effects of WMLs and cerebral atrophy were removed. In addition, executive function assessment was different in the two studies. Digit span backwards and Digit symbol tests were applied in this study. The former reflects working memory performance; the latter requires multiple cognitive abilities including attention, psychomotor speed, complex scanning, visual tracking, and immediate memory [15],[28],[29]. The scores of Digit span backwards and Digit symbol tests were decreased significantly, which means above-mentioned components of executive dysfunction exist in the patients with posterior corona radiata infarction. Besides, increased Trail making B-A and decreased Verbal fluency assessment scores were found, which implies dysfunction of cognitive set shifting, mental flexibility and strategies for word retrieval in these patients.

## Conclusion

Executive dysfunction was observed in the patients with left posterior corona radiata infarction. The axonal loss in the left frontal white matter after stroke was detected by DTI. The abnormalities of MD and FA in the region may be correlated with executive function changes in the patients.

## Abbreviations

ACA: anterior cerebral artery

CT: computed tomography

DIA: luminal diameter

DTI: diffusion tensor imaging

EDV: end diastolic velocity

EPI: echo planar imaging

FA: fractional anisotropy

ICA: iInternal carotid artery

ILA: ischemic leukoaraiosis

IMT: Intima-media thickness

LSD: Least Significant Difference

MCA: middle cerebral artery

MD: mean diffusivity

MFV: Mean flow velocity values

MMSE: Mini-Mental State Examination

MRI: magnetic resonance imaging

PCA: posterior cerebral artery

PSV: peak systolic velocity

TCD: Transcranial Duplex Scanning

T1-FLAIR: T1-weighted fluid attenuatedion inversion recovery

WMLs: white matter lesions

## Competing interests

The authors declare that they have no competing interests.

## Authors’ contributions

CL designed the study, collected the data and wrote the manuscript, CD carried out image post-processing, GL conducted the MR images analysis, LC and JZ assisted with the data collection, JL assisted with the statistical analysis, ZO assisted with the data collection, YZ participated in the design of the study, AX interpreted the results. All authors read and approved the final manuscript.
